# Supplementary material for: Ownership succession, risk taking and debt financing from the perspective of the institutional environment
Source: PLoS One. 2025 Jul 24;20(7):e0327492. doi: 10.1371/journal.pone.0327492 (PMC12289026; doi:10.1371/journal.pone.0327492)
Supplement: Attachment — Submitted filename: Comments.docx [file pone.0327492.s002.docx]

**Comments**

***PLOS ONE***

**Manuscript Number: PONE-D-24-11863**

**Title: Ownership Succession, Risk Taking and Debt Financing from the Perspective of the Institutional Environment**

**General Comment: This paper examines the impact of ownership succession on family firms' debt financing and the mechanisms through which the institutional environment influences this relationship using a sample of Chinese family-controlled listed firms. The authors find that second-generation ownership succession tends to inhibit risk-taking, leading to a reduction in debt financing for the enterprise.**

**Overall, the topic of the paper is interesting and provides various tests. however, I have several concerns that critically limit the contribution of the paper in its current form.**

**1: The discussion of the mediating variable is unclear. The mediating variable cannot be seen from the regression equation. Risk-taking and debt financing are more like two independent dependent variables. So what is the logic between the two? This is the biggest problem of this article.**

**2: In 3.1 Sample selection and data collection, the data selection year is not specified, and it can only be found in the paper conclusion, which needs to be explained clearly in 3.1. Why use the data of these 10 years? Because it is now 2024, the data is obviously a bit outdated.**

**3: Endogeneity problem. Using only lagged terms cannot completely avoid the endogeneity problem. The author needs to add an endogeneity test and select appropriate IV for verification.**

**4: In the empirical part, the institutional variable inhibits the relationship between the two, but from the data test results, its sign is positive, so from the mathematical logic, it should be a positive adjustment. Therefore, when defining the institutional variable, it is necessary to explain whether the larger the data, the better the institutional environment, or vice versa.**

**5: In further research, the author distinguished the institutional environment. What is the necessity for this? The overall index of institutional environment has reflected the differences in different regions. In addition, some regression results are significant while others are not, which economic reasons here are not clearly explained.**
