# Supplementary material for: Ownership succession, risk taking and debt financing from the perspective of the institutional environment
Source: PLoS One. 2025 Jul 24;20(7):e0327492. doi: 10.1371/journal.pone.0327492 (PMC12289026; doi:10.1371/journal.pone.0327492)
Supplement: Attachment — Submitted filename: renamed_d31ef.docx [file pone.0327492.s004.docx]

Response to Reviewer 1 Comments

Thank you very much for taking the time to review this manuscript. According to your suggestions, we have made a substantial revision of the paper from several aspects, such as Mathematical Formulas, Regression Results, and Conclusions, so as to get your approval. And we marked the modified content in yellow. If there is any content that needs to be improved, please contact us. Thank you very much.

**Comments 1:** The discussion of the mediating variable is unclear. The mediating variable cannot be seen from the regression equation. Risk-taking and debt financing are more like two independent dependent variables. So what is the logic between the two? This is the biggest problem of this article.

**Response 1**: 1. With regard to mediating variable, the revised draft adds model (3)，and puts the second- generation ownership, risk-taking and debt financing into the model (3) for regression test of mediating effect.Please see Lines 200-205. In addition, the revised draft also uses the Bootstrap method to test the mediating effect. Please see Lines 338-349 and the Column (1) and (2) of Table 9.

**Comments 2:** In 3.1 Sample selection and data collection, the data selection year is not specified, and it can only be found in the paper conclusion, which needs to be explained clearly in 3.1. Why use the data of these 10 years? Because it is now 2024, the data is obviously a bit outdated.

**Response 2:** The original paper began writing in 2020, when the latest data available was 2019. In addition, the sample size of intergenerational inheritance of Chinese family enterprises before 2008 is very small, and the debt financing of enterprises in 2009 was affected by the 2008 financial crisis, so the data of the original paper chose 2010 to 2019. The revised draft updated the data from 2003 to 2023, and supplemented the reasons for the data selection in the 3.1 Sample selection and data collection of the revised draft.Please see Lines 146-147. In additional,The empirical part of the revised draft also uses new data for analysis.

**Comments 3:** Endogeneity problem. Using only lagged terms cannot completely avoid the endogeneity problem. The author needs to add an endogeneity test and select appropriate IV for verification.

**Response 3:** Thank you for pointing this out. Based on your suggestion, the revised draft adds endogenous test, which is verified by propensity matching score method and instrumental variable method. Please see Lines 290-317

**Comments 4: In the empirical part, the institutional variable inhibits the relationship between the two, but from the data test results, its sign is positive, so from the mathematical logic, it should be a positive adjustment. Therefore, when defining the institutional variable, it is necessary to explain whether the larger the data, the better the institutional environment, or vice versa.**

**Response 4: In the empirical part, from the data test results, the symbol of the coefficient of the interaction between institutional environment and second-generation ownership is positive. Whether the regulatory effect of the institutional environment is positive or negative needs to be considered in combination with the relationship between second-generation ownership and debt financing in the previous benchmark regression. The coefficient of second-generation ownership in the previous benchmark regression is negative. Therefore, the better the institutional environment, the negative relationship between second-generation ownership and debt financing is weakened, or vice versa. Please see Lines 350-363.**

**Comments 5: In further research, the author distinguished the institutional environment. What is the necessity for this? The overall index of institutional environment has reflected the differences in different regions. In addition, some regression results are significant while others are not, which economic reasons here are not clearly explained.**

**Response 5: In further research, the paper distinguishes the specific elements of the institutional environment, because the score of the institutional environment is calculated by the scores of these specific environmental elements. The paper hopes to explore the specific impact of various environmental factors on the debt financing of family enterprises in the intergenerational inheritance period, in order to put forward more targeted suggestions. In addition, some regression results are significant, while others are not, and the possible reasons are given in the revised draft.Please see Lines 399-405.**
