# Supplementary material for: Ownership succession, risk taking and debt financing from the perspective of the institutional environment
Source: PLoS One. 2025 Jul 24;20(7):e0327492. doi: 10.1371/journal.pone.0327492 (PMC12289026; doi:10.1371/journal.pone.0327492)
Supplement: Attachment — Submitted filename: R-20250425.doc [file pone.0327492.s005.doc]

Reviewer #2: Overall, it is a very good study, however, following points can be considered for further development:

1. The research gap needs to be strengthened.

Answer：The author has reorganized the literature on debt financing in intergenerational inheritance of family businesses, and compared in detail the viewpoints, methods, and conclusions of different previous studies, pointing out the shortcomings of previous research. Please refer to lines 39-64 for details

2. The introduction section needs to be strongly built.

Answer：The author added institutional and practical backgrounds related to the research in the introduction, pointed out the shortcomings of previous research, and introduced research questions around agency theory. Please refer to lines 21-30, 39-64, and 65-69 for details.

2. The authors should use theory to build the framework of the study. Agency theory may be more relevant.

Answer：The author has used agency theory to reconstruct the research framework：in the introduction section of the paper, agency theory is introduced to propose research questions. In the hypothesis proposal section, logical reasoning is carried out based on agency theory. In the conclusion and discussion section, the agency theory is used to discuss the research results. Please refer to lines 65-69, 89-117, 121-142, 161-182, and 465-505 for details.

3. Hypotheses formulation needs to be logical in the light of theory used.

Answer：The author used agency theory to conduct a logical reasoning of the hypotheses again. Please refer to lines 89-117121-142 and 161-182 for details.

4. Why sample started from 2003? This needs to be justified.

Answer：The author provided additional explanations on why sample started from 2003. Please refer to lines 191-198 for details.

5. The results need to be discussed in the light of theory and in the light of previous studies.

Answer：The author has conducted a new discussion on the research conclusions based on agency theory and previous studies. Please refer to lines 465-482 for details.

6. Theoretical and practical implications needs to be further strengthened.

Answer：The author provided a new exposition on the theoretical and practical significance at the end of the paper. Please refer to lines 483-505 for details.
